# Single-Access Laparoscopic Rectal Surgery Is Technically Feasible

**DOI:** 10.1155/2013/687134

**Published:** 2013-03-20

**Authors:** Siripong Sirikurnpiboon, Paiboon Jivapaisarnpong

**Affiliations:** Colorectal Division, General Surgery Department, Rajavithi Hospital, Rangsit University, Bangkok 10400, Thailand

## Abstract

*Introduction*. Single-access laparoscopic surgery (SALS) has been successfully introduced for colectomy surgery; however, for mid to low rectum procedures such as total mesorectal excision, it can be technically complicated. In this study, we introduced a single-access technique for rectum cancer operations without the use of other instruments. *Aims*. To show the short-term results of single-access laparoscopic rectal surgery in terms of pathologic results and immediate complications. *Settings and Design*. Prospective study. *Materials and Methods*. We selected middle rectum to anal canal cancer patients to undergo single-access laparoscopic rectal resection for rectal cancer. All patients had total mesorectal excisions. An umbilical incision was made for the insertion of a single multichannel port, and a mesocolic window was created to identify the inferior mesenteric artery and vein. Total mesorectal excision was performed. There were no perioperative complications. The mean operative time was 269 minutes; the median hospital stay was 7 days; the mean wound size was 5.5 cm; the median number of harvested lymph nodes was 15; and all patients had intact mesorectal capsules. *Statistical Analysis Used*. Mean, minimum–maximum. *Conclusion*. Single-access laparoscopic surgery for rectal cancer is feasible while oncologic principles and patient safety are maintained.

## 1. Introduction

Single-access laparoscopic surgery (SALS) has been successfully introduced for colectomy [1]. But for mid to low rectum procedures, such as total mesorectal excision, it can be technically complicated. Only a few reports have been published about single-access laparoscopic low anterior resection [2–6]. The usual techniques used to maintain an adequate operative field for TME are lifting the rectum with a second forceps or suspending the rectum with transparietal sutures. In this study, however, we introduced a single-access technique for rectal surgery without the use of other instruments.

## 2. Materials and Methods

The study took place from December, 2011 to December, 2012 in the Tertiary Care Unit of Rajavithi Hospital. All operations were performed by a colorectal surgeon. 

The inclusion criteria were (1) patients who had been diagnosed with cancer at the middle or low rectum or the anal canal and (2) patients who had rejected neoadjuvant chemotherapy.

The exclusion criteria were (1) patients who were unfit for surgery; (2) patients who did not attend for followup; (3) patients for whom anesthesia was contraindicated; and (4) patients with asymptomatic stage IV disease.

The study was approved by the Ethical Committee of Rajavithi Hospital.

## 3. Operative Technique

All the procedures were performed by the same colorectal surgeon. All of the patients underwent bowel preparation 1 day preoperatively either with 4 litres of polyethylene glycol electrolyte solution or 90 mL of sodium phosphate solution depending on their comorbid disease.

**Figure 1:**
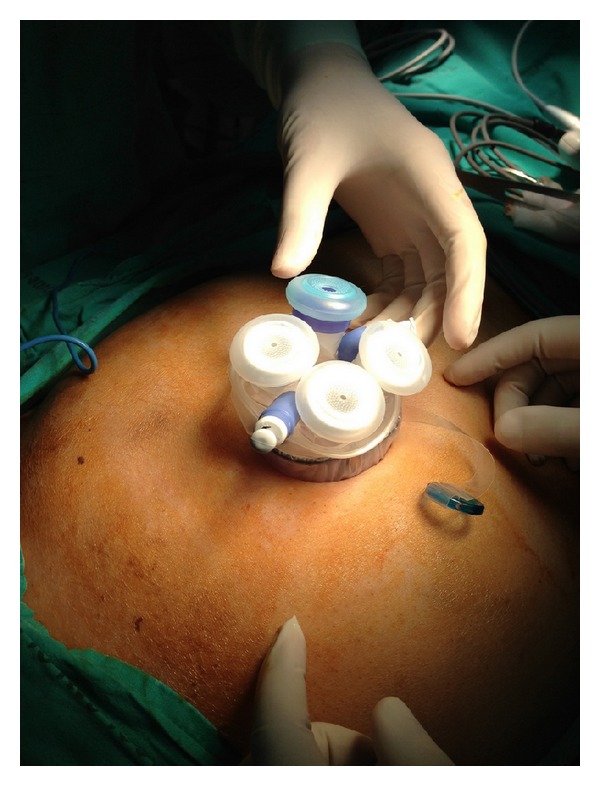
Port position.

Surgical procedures were performed through a 5-6 cm single umbilical incision using a single-access multiport device (Glove Port-Single Port, Nelis Ltd., Gyeonggi-do, Korea) (Figure 1) that allows three additional trocars (two 5 mm and one 10–12 mm) to be inserted and has a CO_2_ connection for insufflations (Figure 1). The camera was a flexible videolaparoscope (Olympus Medical Systems Corp., Tokyo, Japan).

The reverse Trendelenburg semiright lateral position was used. The surgeon and cameraman stood on the right side of the patient.

**Figure 2:**
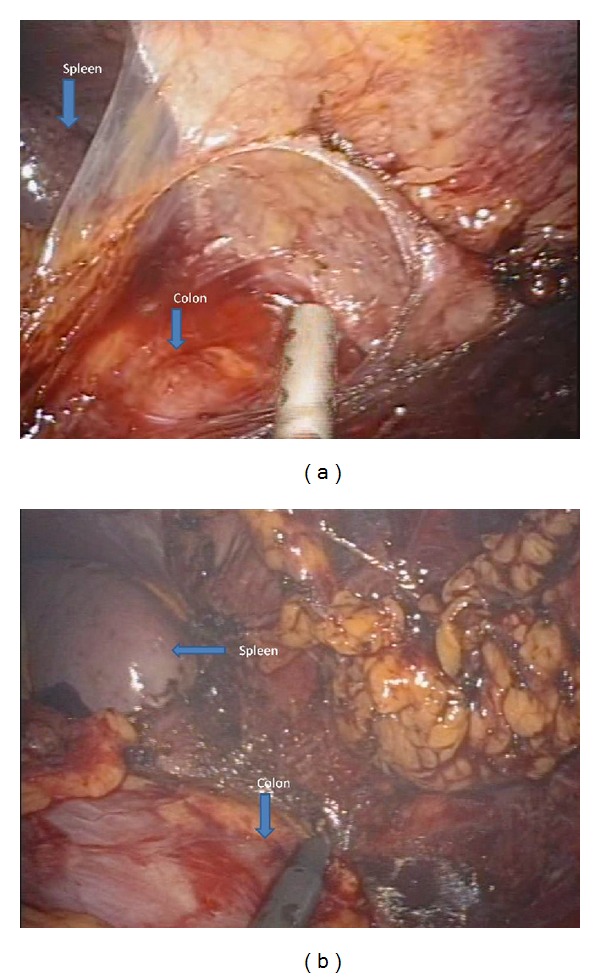
Splenic flexure mobilization.

**Figure 3:**
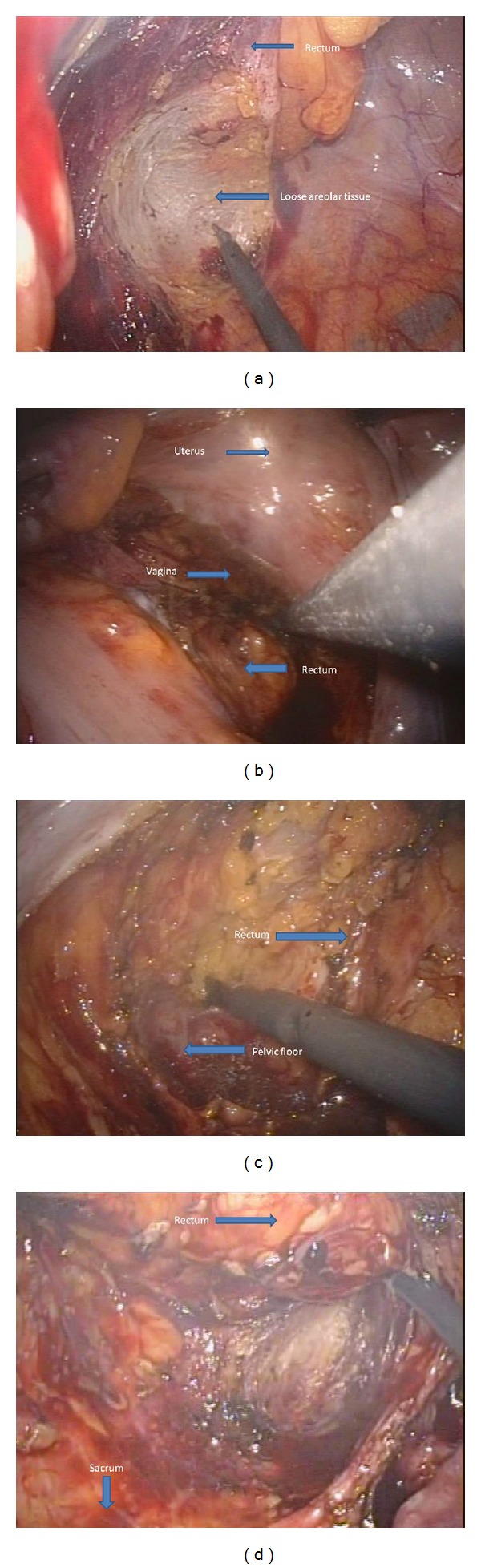
Pelvic dissection.

**Figure 4:**
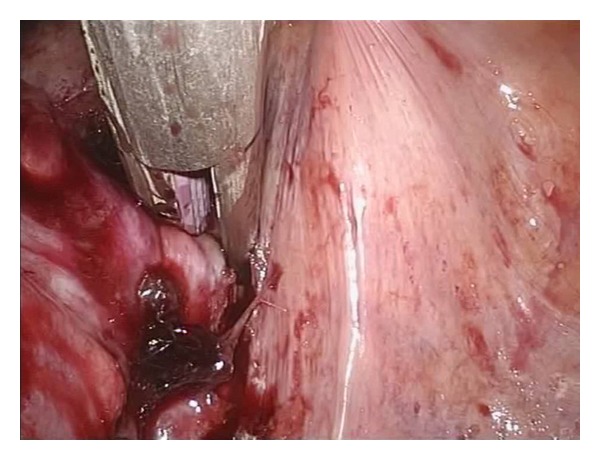
Position of placed Endo articulating linear stapler.

Operations were performed using a surgical technique similar to the standard laparoscopic (medial-to-lateral) approach. The inferior mesenteric artery and the inferior mesenteric vein were both skeletonized and clipped by Hem-o-lok (Teleflex Medical, Durham, NC, USA) or Liga clip (Johnson and Johnson, New York, NY, USA) and divided with scissors. Then, we dissected downwards in a semicircular motion from the mesenteric window to the pelvis on the right side of the rectum. For posterior dissection, the rectum was grasped and pushed anteriorly using Endo grasp forceps or a flexible Endo clinch and dissection was performed from the promontory of the sacrum in a semicircular motion deep down to the coccyx. The next step was to mobilize the sigmoid colon up to the splenic flexure. The descending colon was grasped by Endo grasp forceps or flexible Endo clinch and pulled anteromedially to clearly identify the lateral peritoneal attachment, and it was then severed by cauterization up to the splenic attachment (Figure 2). At this point downward, medial traction was applied to the colon to expose the splenic attachment and then divided with cautery. The flexible tip videolaparoscope proved helpful for changing the angle and operative view in this phase. To facilitate the process of dissecting deep into the pelvis, we used the force of gravity by moving the patient into the reverse Trendelenburg position, and we also utilised a port that allowed two Endo grasps or Endo clinches to push the rectum anteriorly. For anterior dissection, the peritoneal attachment was pulled up anteriorly, and the mobilized rectum was dissected (Figure 3). In the low anterior resection, the rectum was transected using 2 endoscopic linear staplers (Endo GIA, Covidien plc, Dublin, Ireland). The position of the applied stapler is shown in Figure 4. Due to limitations in Endo stapler angulation and pelvis diameter, the proximal colon was extracted through the umbilical incision. Resection was achieved following extracorporealization, and anastomosis was performed with the double stapling technique using a transanally inserted circular stapler (CDH29, Ethicon Endo-Surgery Inc., Cincinnati, OH, USA). Diverting stoma was not usually performed. A pelvic drainage tube was inserted at a new stab incision at the right lower quadrant under laparoscopic view. In the APR cases, we started the perineal resection phase after finishing the intraperitoneal phase using the standard AP resection technique. In our hospital, cylindrical abdominoperineal resection is not routinely used.

## 4. Data Collection

Demographic data including patients' age, gender, and body mass index (BMI) were tabulated together with their history of prior abdominal surgery. Intraoperative parameters including operative time, estimated blood loss, and intraoperative complications were analyzed. 

Pathologic characteristics such as depth invasion, lymph node retrieval, circumferential margin, distal margin, and mesorectal capsule status were reviewed, and postoperative outcomes including length of stay in hospital and complication rates were collected.

## 5. Results

Between December, 2011 and December, 2012, 10 patients (4 females and 6 males, mean age 69 years, range 52–86) underwent SALS for middle rectal, low rectal, and anal canal cancer. The operations comprised 9 abdominoperineal resections and 1 low anterior resection. All patients had stage II or III disease preoperatively. None received preoperative neoadjuvant therapy because they had rejected it. The average body mass index was 21.77 (range 15 to 30 kg/m^2^) (Table 1). In all cases, the patients' consent for single-access laparoscopic surgery was obtained.

The median total surgical time was 269 minutes (range 200–300 min). The average intraoperative blood loss was 145 mL (range 50–300 mL). In the LAR case, the anastomosis was 6 cm from the anal verge (Table 2). Intraoperatively, there were no complications, but postoperatively, there were 6 problems: 2 cases of lung atelectasis; 2 instances of nonorganic cause delirium; 1 case of thrombophlebitis on the forearm; and 1 case of perineal wound infection. None of the patients developed neurogenic bladder (Table 3), and none of the male patients developed any sexual disorders.

**Figure 5:**
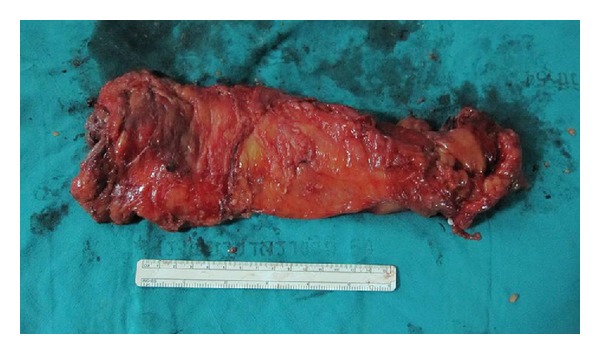
Specimen in LAR.

**Figure 6:**
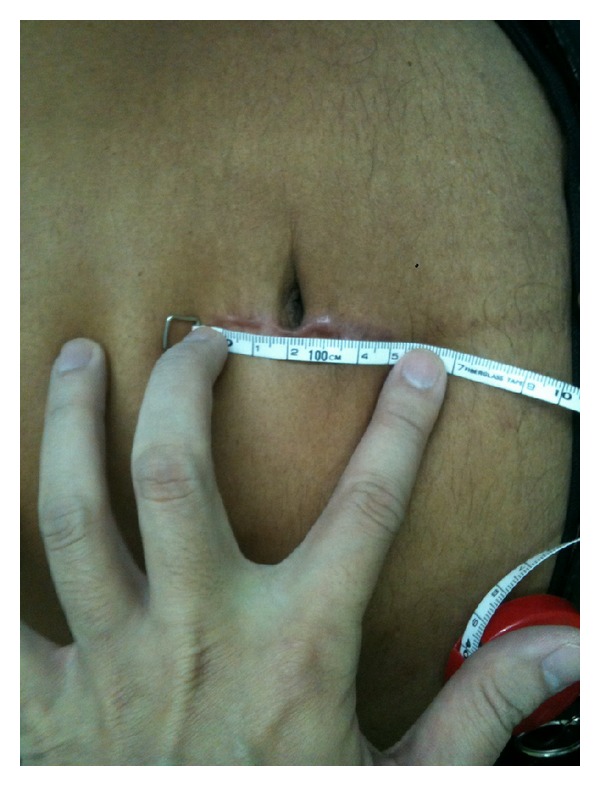
Postoperative wound length.

The median number of harvested lymph nodes was 15 (range 8–30 nodes). Postoperatively, all patients were oncologic stage II or III (4 patients were stage II, and the other 6 were stage III), and all patients received adjuvant chemoradiation therapy. Surgical margins were negative in all patients, with a distal margin of at least 2 cm and circumferential margin of at least 2 mm in all cases (Figure 5). And the mean wound size was 5.5 cm (Figure 6). All patients were allowed oral fluid on the first postoperative day; bowel movement median occurrence was on the third postoperative day; free light diet was allowed on the subsequent day; and patients were discharged when they were able to return to a regular diet with the exception of one patient who developed a perineal wound infection. He was discharged on postoperative day 10 in good condition. There were no readmissions postoperatively.

## 6. Discussion

Nowadays, the use of minimally invasive surgery is widely accepted. NOTES (natural orifice translumenal endoscopic surgery) and SALS are at the cutting edge of these techniques. SALS has some significant advantages over NOTES, in particular its facilitation of the use of all common laparoscopic instruments such as laparoscopes, straight and articulating instruments, and the full range of commercially available energy-based dissecting devices [13]. The first report of single-access laparoscopic surgery was a right hemicolectomy in 2008 [14]. Recently, a report from Egi et al. [15] showed no difference in oncologic results between single-port laparoscopic techniques and conventional ones. However, the major problem from a surgical point of view is that the concept of “triangulation,” to which laparoscopic surgeons have grown accustomed to in terms of both the instruments and scope, is lacking [16]. Examples of this are the laparoscope's view and articulating instruments. With regard to rectum surgery, the major technical problems are (1) the difficulty in obtaining TME and (2) the limitations of Endo staple instrument use in the pelvis.

A report from Leroy et al. [17] showed that laparoscopic surgery achieved good long-term oncologic results in TME. In single-access laparoscopic surgery, the first report from Hamzaoglu et al. [9] shows promising preliminary pathologic results in 4 cases of LAR with the introduction of a sutured sigmoid hung into the abdominal wall as a way of attaining adequate exposure for TME. In 2010, Uematsu et al. [18] reported a novel single-access port for use in a sigmoidectomy, and in 2011 there was a report of the use of a suspending bar to lift up the sigmoid for TME [10] with excellent pathologic results. Another 2 reports [11, 12] also showed good pathologic results (Table 4). Our study attempted to share our initial experience of performing single-port laparoscopic surgery of rectal cancer in which we achieved equally good pathologic results. From our results, we believe that (1) a bigger port was helpful in reducing instrument collision during operations and enlarged the working channel to manipulate operative field; (2) articulating instruments, especially Endo clinches or graspers, are useful as they help to maintain “triangulation”; (3) a flexible videolaparoscope is necessary or even essential because of its adjustable tip which helps to provide an adequate operative field in rectal dissection; and lastly (4) the reverse Trendelenburg position is useful in helping to pull the rectum in a cranial direction using the force of gravity. With regard to the pelvic diameter and the limited articulation of Endo linear staplers, we had only limited experience; however, Kim et al. [19] reported that the use of multiple stapler firings was a significant risk factor for anastomotic leakage, and they concluded that a reduction in the number of linear stapler firings is necessary to avoid anastomotic leakage after laparoscopic colorectal anastomosis with a double stapling technique. In the LAR case in our study, we used 2 laparoscopic staples to transect the rectum vertically, and we did not create a protective ileostomy.

## 7. Conclusion

The single-access laparoscopic technique is gaining favour with surgeons around the world with the evolution of minimally invasive techniques and instruments. Our results show that the single-access technique for rectal surgery seems to be safe and effective with potentially reproducible oncologic results. In the future, randomized clinical trials should be carried out to confirm our preliminary results showing the benefits of single-access procedures.

## Figures and Tables

**Table 1:** Demographic data.

Age (mean, years)	69 ± 11.76 (52–86)
BMI (mean, min–max)	21.77 ± 4.48 (15.00–30.00)
Sex (male/female)	6/4
ASA classification (median, min–max)	2 (1–3)
Location of tumor	
Anal canal/lower rectum	9
Middle rectum	1
Clinical stage	
Stage II	2
Stage III	8

**Table 2:** Operation and pathologic result.

Operation	
APR	9
LAR	1
Surgical time (minutes)	269 ± 41.75 (200–300)
Blood loss (mL)	145 ± 76.19 (50–300)
Pathologic result	
T stage	T3—7 patients T2—3 patients
Lymph node retrieval (median, min–max)	15 (8–30)
Quirk mesorectal grading [7, 8]	Grade 3—9 patients Grade 2—1 patients
CRM	All negative
Pathologic staging	
Stage II	4
Stage III	6

**Table 3:** Postoperative details and complications.

Immediate postoperative complication	
Postoperative lung atelectasis	2
Perineal wound infection	1
Thrombophlebitis	1
Postoperative delirium	2
Hospital stay (day) (median, min–max)	7 (5–10)
30-day mortality	0
Postoperative first bowel movement (day) (median, min–max)	3 (2-3)
Port site wound length (cm) (mean, range)	5.5 ± 0.44 (5-6)

**Table 4:** Previous results in Single access rectal cancer surgery.

Author, year	Patient number	Operation	Special Technique or Instrument	Port type	Mean operative time (minutes)	Staging	Mean wound length	Quirke's mesorectal fascia grade
Hamzaoglu et al., 2011 [9]	4	3 LAR 1 TAE	Suture-hung sigmoid with abdominal wall	Triport	347	2 stage III 2 stage I	3.5 cm	3
Uematsu et al., 2011 [10]	7	LAR	Suspending bar and extracorporeal magnet	Self innovation	205	2, stage II 5 stage III	3 cm	NA
Hirano et al., 2012 [11]	15	AR	NA	EZ lap protector + 12 mm port	276	0 stage 0 3 stage I 3 stage II 7 stage III 2 stage IV	2.8 cm	NA
Hua-Feng et al., 2012 [12]	20	APR	Start from perineal resection phase	Self-innovation	138	NA	NA	NA

LAR: low anterior resection, TAE: transabdominal anal excision, AR: anterior resection, APR: abdominoperineal resection.
